# *Echis ocellatus* Venom-Induced Reproductive Pathologies in Rat Model; Roles of Oxidative Stress and Pro-Inflammatory Cytokines

**DOI:** 10.3390/toxins14060378

**Published:** 2022-05-29

**Authors:** Babafemi Siji Ajisebiola, Priscilla Ifeoluwa Alamu, Adewale Segun James, Akindele Oluwatosin Adeyi

**Affiliations:** 1 Department of Zoology, Osun State University, Osogbo 230232, Nigeria; priscillaalamu1@gmail.com; 2 Biochemistry Program, Department of Chemical Science, Augustine University, Lagos 1010, Nigeria; adewale.james@augustineuniversity.edu.ng; 3 Animal Physiology Unit, Department of Zoology, University of Ibadan, Ibadan 200005, Nigeria

**Keywords:** reproduction, *Echis ocellatus*, cytokines, sex hormones, envenoming, oxidant damage

## Abstract

This study reported reproductive pathologies associated with *Echis ocellatus* venom in animal model. Twenty male Wistar rats with body weight between 180 and 220 g were selected randomly into two groups (n = 10). Rats in group 1 served as the control while rats in group 2 were envenomed with a single intraperitoneal injection of 0.055 mg/kg^−1^ (LD_6.25_) of *E. ocellatus* venom on the first day and a repeated dose on the twenty fifth day. Both control and envenomed rats were monitored for fifty consecutive days. The venom caused a significant (*p* < 0.05) reduction in sperm motility, count, and volume, with increased sperm anomalies in envenomed rats compared to the control. Likewise, serum concentrations of male reproductive hormones were significantly (*p* < 0.05) higher in envenomed rats. Increased levels of malondialdehyde were accompanied by a significant (*p* < 0.05) decrease in reduced glutathione and catalase activity in the epididymis and testis tissues of envenomed rats. The venom enhanced the release of epididymal and testicular tumor necrosis factor-alpha and interleukin1-beta compared to the control. Furthermore, severe pathological defects were noticed in tissues of the testis and epididymis of envenomed rats. This study demonstrated that *E. ocellatus* venom toxins can induce reproductive dysfunction in male victims of snake envenoming.

## 1. Introduction

Reproductive dysfunction is a malady of the reproductive system that has evolved as a serious global public health challenge [1,2]. Reproductive dysfunction resulting from pathological conditions in males is a complex physiological process that is associated with different risks and pathological mechanisms that could directly or indirectly affect male reproductive functions and predispose to the development of infertility [3]. Globally, male reproductive infertility has been a significant health problem since the mid-1950s which is on the rise and seriously becoming a public health concern in this age, attracting the focus of clinicians and researchers in recent decades [1,4].

Researchers have documented several factors that may be liable for the impairment of reproductive function in males, which may be psychological resulting from stress or trauma, pathological due to inflammation or infection, physiological as a result of body constitution or age and lifestyle, such as alcohol drinking or drug abuse, including environmental factors, arising from exposure to heavy metals or natural toxins etc. [5,6,7]. However, the impact of natural toxins produced by animals on reproductive functions has received less attention despite well established reports that toxins from venom producing animals, such as bees and snakes, have detrimental effects on male reproductive physiology [8,9]. However, quite a number of studies have reported various pathophysiological conditions caused by venom of some snake species on male reproductive functions in animal models. Venoms of *Crotalus durissus* ssp. and *Daboia russelli* have been documented to cause elevated sperm anomalies and atrophy in testis after envenomation respectively [10,11], while *Bothrops jararaca* venom toxins have been reported to induce inhibition of spermatogenesis [12].

Reports abound that the mechanism underlying male reproductive toxicity could be attributed to an increase in oxidative stress (OS) markers resulting from excessive generation of reactive oxygen species (ROS), reproductive hormonal imbalances arising from disruption of endocrine secretions and alteration in expression pattern of genes responsible for spermatogenesis, as well as exerting epigenetic effects in the reproductive potency of the offspring [13]. However, OS denotes a familiar mechanism in the foreknowledge of male reproductive dysfunction and presents a distinctive explanation to the majority of male infertility occurrences [14]. Furthermore, most cases of male sexual dysfunction complexly link OS and institute malicious pathways that undermine the structure and functions of male reproductive tissues [15]. In fact, about 40–50% of the occurrences of disordering in male fertility parameters are considered linked to mechanisms interconnected with OS [16]. Moreover, studies have demonstrated that cytokines recognized as mediators of oxidant damage may concurrently alter sperm quality and male reproductive system as functions of some cytokines are dependent on their concentration in male fertility [16]. It has been reported that some specific proinflammatory cytokines are higher in infertile males, and their elevated concentrations are frequently connected with a reduction in semen quality [17].

*Echis ocellatus* (Africa saw-scaled viper) is a poisonous specie of snake known to account for serious economic and general health challenges in tropical regions of the world, most especially in West Africa [18]. Moreover, the species is considered to be of high medical importance and its envenoming could be life threatening if immediate proper treatment is not administered [18]. According to reports, cases of human casualties resulting from *E. ocellatus* envenoming are higher when compared to other African snakes combined, with a record of 90% of bites and over 60% of mortalities, including several thousand permanent disabilities [19,20]. In Nigeria, close to 90% of incidences of snakebites are attributed to *E. ocellatus* envenoming with 60% of deaths due to severe fatalities after envenoming while others have suffered severe disabilities of various degrees [21]. 

The use of conventional antivenom remains the only effective treatment to snakebite envenoming but is faced with several drawbacks, such as high cost, scarcity, and the combined inability to overturn injuries actuated by the venom on tissues of organs in envenomed victims [22]. In rural communities of most parts of the world, victims of snakebites are mostly farmers, agricultural workers, nomads, and rural dwellers who seek alternative treatment using traditional methods due to unaffordability and inaccessibility to antivenom treatment [18,21]. However, most of these alternative snakebite treatments are not reliable, but may only lessen the impact and not effectively neutralize the actions of the venom toxins after envenoming. Consequently, the resulting effects may have detrimental implications on different tissues of the body, which may cause dysregulation in physiological functions in envenomed victims. These factors necessitate scientific investigations to unravel pathologies on vital reproductive organ functions of envenomed victims that may be connected with snakebite envenoming when proper treatment is not administered. 

Venom of snakes are modified saliva composed of mixtures of different bioactive proteins, polypeptides, and chemicals with significant toxic effects on biological systems [23]. Viper venoms are very toxic and pathophysiological alterations in system functions resulting from *E. ocellatus* envenoming have been well documented [24]. Major toxins present in *E. ocellatus* venom that are responsible for toxic actions after envenoming include snake venom metalloproteinases (SVMPs) and phospholipase A2, which possesses hemorrhagic, cytotoxic, myotoxic, neurotoxic, cardiotoxic, and anticoagulant effects [24,25]. Based on our findings, there are limited toxicological studies that evaluate the impact of snake venom toxins on the reproductive system combined with a paucity of information regarding the impact of viper venom on reproductive functions. The study on pathological conditions of male reproductive system is essential so as to unravel reproductive inefficiency that may arise in envenomed patients. Accordingly, this current study aimed to broaden scientific knowledge on the pathological effects of viper envenoming on male reproductive physiology by accessing the roles of oxidative stress and elevated pro-inflammatory cytokines in *E. ocellatus* envenomed male rats. 

## 2. Results

### 2.1. Clinical Signs of Toxicity

The envenomed rats showed several clinical signs of toxicity, including dizziness, sluggish movements, decrease in food and water consumption, and mild heamorrhage noticed at the site of venom injection. Also, one rat died on the 19th day of the experiment in the envenomed group. However, these clinical signs of toxicity were absent in control rats (Table 1). Body weight gain of the control was significantly (*p* < 0.05) higher compared to the envenomed rats. Likewise, the testicular weight and testiculo somatic index of envenomed rats significantly (*p* < 0.05) decreased compared to the control group (Table 2). 

#### 2.1.1. Epididymal Sperm Parameters

A significant (*p* < 0.05) decline was noticed in the percentage of motile spermatozoa in envenomed rats compared to the control, resulting in a significant (*p* < 0.05) increase in the percentage of immotile spermatozoa in envenomed rats (Table 3). Moreover, sperm count and volume showed a significant (*p* < 0.05) reduction in rats injected with venom when compared to control (Table 3).

#### 2.1.2. Sperm Abnormalities in Envenomed Rats

The venom showed various toxic effects on sperm morphology in the envenomed rats. The percentage of sperm abnormalities in envenomed rats was significantly (*p* < 0.05) higher compared to the control. Higher abnormalities of sperm cells with banana shape, folded sperm, no hook, and wrong-angled hook were noticed in the envenomed rats and values obtained were significantly (*p* < 0.05) different compared to the control. Unique sperm abnormalities not found in the control but present in envenomed rats are abnormal mid-piece, double tail, and double head (Table 4). 

### 2.2. Reproductive Hormone Concentrations

The levels of male sex hormones in the serum of experimental rats are presented in Table 5. There was a significant (*p* < 0.05) elevation in the concentration of testosterone, follicle stimulating hormones (FSH), and luteinizing hormone (LH) in serum of envenomed rats when compared to the control. 

### 2.3. Oxidative Stress Parameters 

**Figure 1:**
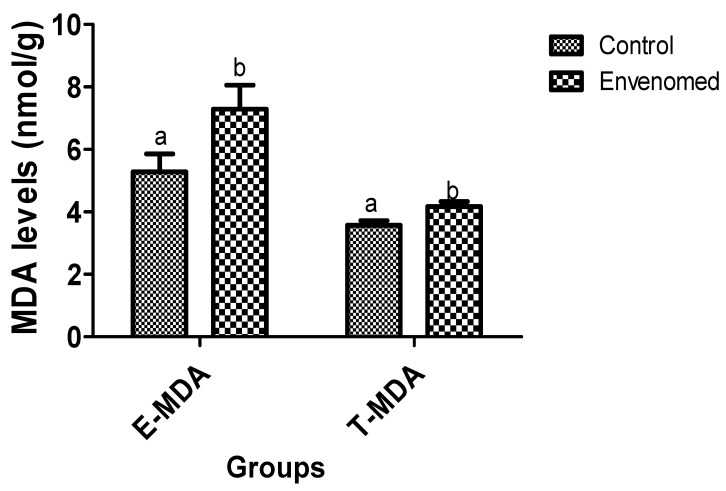
Level of malondialdehyde in epididymis and testis tissues after *E. ocellatus* envenomation. Data are expressed as mean ± standard error, (n = 5). Values with the same superscript are considered not significant (*p* < 0.05). E-MDA: Epididymis Malondialdehyde, T-MDA: Testis Malondialdehyde.

**Figure 2:**
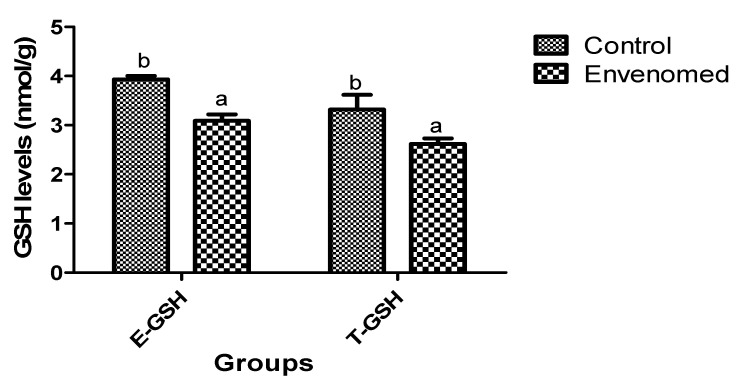
Glutathione concentration in epididymis and testis tissues after *E. ocellatus* envenomation. Data are expressed as mean ± standard error, (n = 5). Values with the same superscript are considered not significant (*p* < 0.05). E-GSH: Epididymis Glutathione, T-GSH: Testis Glutathione.

**Figure 3:**
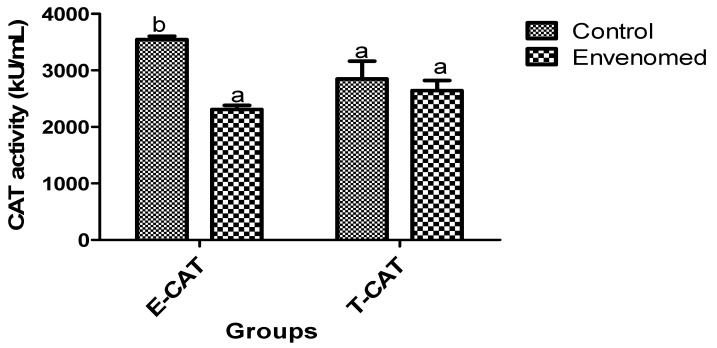
Activity of catalase in epididymis and testis tissues after *E. ocellatus* envenomation. Data are expressed as MEAN ± S.E, (n = 5). Values with the same superscript are considered not significant (*p* < 0.05). E-CAT: Epididymis Catalase, T-CAT: Testis Catalase.

The results of oxidative stress status of the envenomed rats showed that levels of malondialdehyde (MDA) in testis and epididymis tissues of envenomed rats were significantly (*p* < 0.05) higher compared to the control (Figure 1). On the other hand, there was a significant (*p* < 0.05) reduction in levels of glutathione in testis and epididymis tissues of the envenomed compared to the control (Figure 2). Moreover, the activity of catalase (CAT) in epididymis tissue of envenomed rats was significantly (*p* < 0.05) lower compared to the control. However, there was no significant (*p* < 0.05) difference in the activity of CAT in the testis of control and envenomed rats, but a marked increase in CAT activities was recorded in control when compared to the envenomed rats (Figure 3)

### 2.4. Pro-Inflammatory Cytokines Production

**Figure 4:**
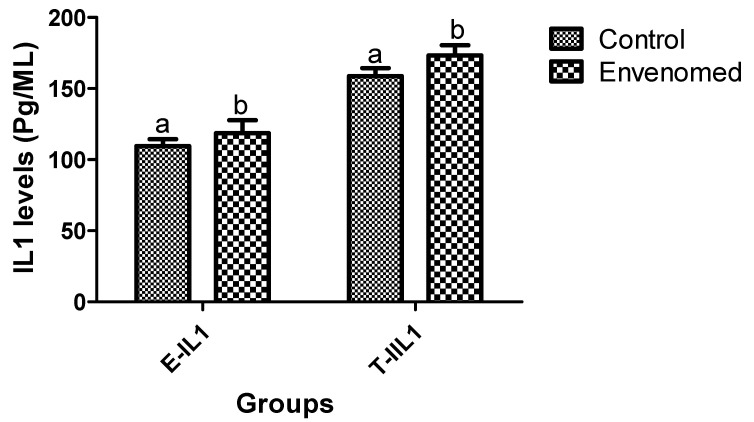
Interleukin1-Beta (IL-1β) responses in epididymis and testis tissues after *E. ocellatus* envenomation. Data are expressed as mean ± standard error, (n = 5). Values with the same superscript are considered not significant (*p* < 0.05). E-IL1: Epididymis Interleukin1-Beta, T-IL1: Testis Interleukin1-Beta.

**Figure 5:**
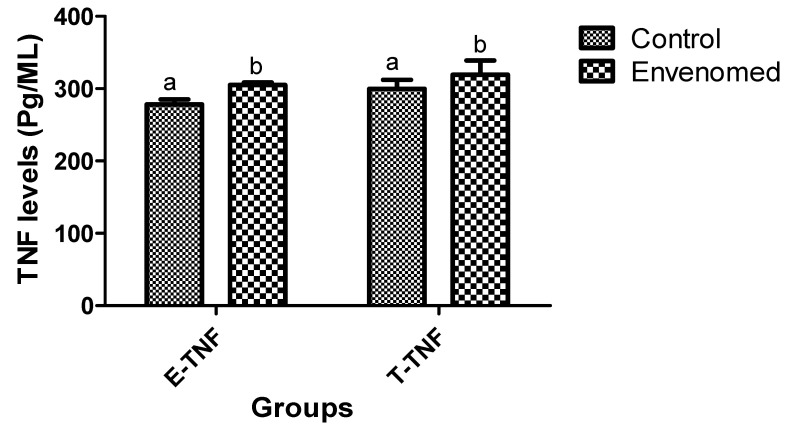
Tumor necrosis factor-alpha (TNF-α) responses in epididymis and testis tissues after *E. ocellatus* envenomation. Data are expressed as mean ± standard error, (n = 5). Values with the same superscript are considered not significant (*p* < 0.05). E-TNF: Epididymis Tumor necrosis factor-alpha, T-TNF: Testis Tumor necrosis factor-alpha.

The venom significantly (*p* < 0.05) up-regulated interleukin1-beta (IL-1β) production in epididymis and testis tissues of envenomed rats compared to the control (Figure 4). Likewise, there was a significant (*p* < 0.05) elevation of tumor necrosis factor-alpha (TNF-α) responses in the epididymis and testis tissues of envenomed rats when compared to the control (Figure 5). 

### 2.5. Histopathology

**Figure 6:**
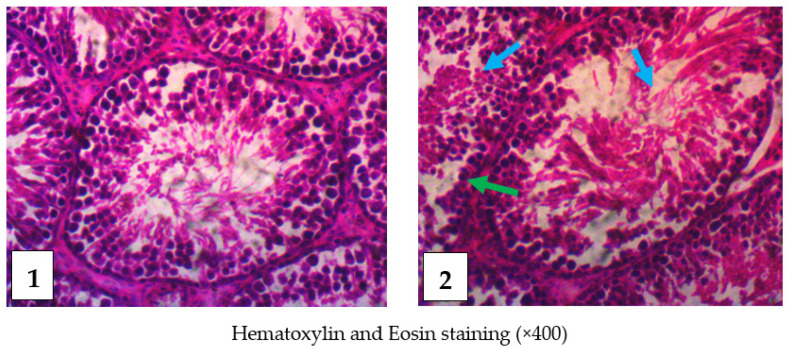
Histological examination of the testis. Control (**1**): The slides of control showed no observable lesion. The seminiferous tubules was observed closely packed and showed uniformly sized with numerous spermatogenic cells. Envenomed (**2**): The slides of the envenomed rats revealed inflammatory response in the dermis, tubular atrophy, degeneration of the seminiferous tubules with necrotic spermatogenic cells (blue arrows), and distortion of germinal epithelium (green arrow).

**Figure 7:**
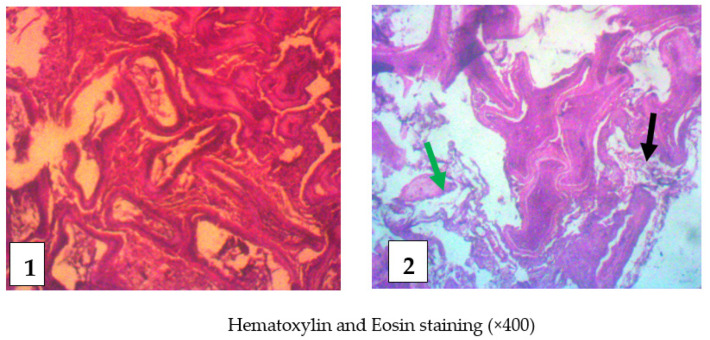
Histological examination of the epididymis Control (**1**): Showed a normal cauda with no observable lesion, Envenomed (**2**): Inflammatory infiltrate in the cauda (black arrows) and atrophy of the tubules and accentuation of interstitium (green arrow).

The slides of testis tissues of control rats showed a closely packed seminiferous tubules with no observable defect, whereas testis tissues of envenomed rats revealed an inflammatory response in the dermis, a distorted germinal epithelium, degeneration of the seminiferous tubules, and tubular atrophy (Figure 6). Moreover, histological examination of epididymis tissues of envenomed rats revealed inflammatory infiltrate in cauda, atrophy of the tubules, and accentuation of interstitium, while epididymis tissue of the control showed a normal cauda with no observable lesion (Figure 7). 

## 3. Discussion

The venom of *E. ocellatus* contains numerous toxic enzymes, majorly snake venom metalloproteinases (SVMPs) that interfere with different biological systems of the body, eliciting severe clinical complications in envenomed victims [25]. This study assessed the toxicological effects of *E. ocellatus* venom on reproductive functions initiated through the induction of oxidative stress (OS) and modulation of pro-inflammatory cytokines in testis and epididymis of rats injected with the venom. Findings from this study revealed that *E. ocellatus* venom caused a significant reduction in body weight, testicular weight, and organo-somatic index compared to the control, suggesting that the venom may have a direct interaction with the testicular tissues resulting in testicular toxicity. Studies have reported such an observation in the testis of mice after exposure to toxicants [26]. 

Spermatozoa matures in the epididymis with some required processes, including sperm plasma membrane remodeling, membrane protein reordering, enzyme modification, and nuclear reconfiguration [27]. In this study, *E. ocellatus* venom caused a significant reduction in the epididymal sperm motility, sperm count, and volume in the envenomed rats which may be due to OS induction in the epididymis as there was a significant increase in levels of OS biomarker; malonaldehyde in testicular and epididymal tissues of the envenomed rat. In addition, the epididymis possesses hormonally sensitive tissues that may undergo changes due to effects of toxicants and become senescent [28]. Pasqualotto et al. [28] had suggested that epididymal senescence may lead to a decrease in sperm motility. The resultant increase in immotile sperms with a decline in sperm count and volume post envenomation in rats is a consequence of oxidative damage resulting from enhanced ROS levels in sperm plasma membrane [29]. Normal cell activity must be protected by ceaseless elimination of surplus ROS, and in situations where the antioxidant defence system is overpowered due to a surge in production of free radicals or the endogenous antioxidant capacity is overwhelmed by elevated levels of seminal plasma ROS, the consequence may be detrimental to semen parameters [30], as observed in this study. However, such physiological defects on sperm cells have been reported using a viper venom [12]. 

In this present study, *E. ocellatus* venom caused a significant increase in sperm abnormalities and OS factor could be responsible for the induction of sperm anomalies as levels of lipid peroxidation (LPO) significantly increased, resulting in the depletion of glutathione (GSH) and catalase contents in the testis and epididymis of envenomed rats. GSH is a defensive mechanism for sperm cells, eliminating free radicals using its free sulphydryl class through direct interplay with ROS, and sperm plasma membranes are protected from cytotoxic aldehydes released during LPO by the direct reaction of GSH present in the extracellular space [31]. On the other hand, LPO is known to cause the quick depletion of adenosine triphosphate (ATP) in sperm cells, leading to reduction, impaired motility, disruption in acrosome membrane, and depletion in potency to fuse with the ovum [31]. According to Atig et al. [32], defective motility of spermatozoa as a result of instability of the mid-piece is a consequence of LPO of the sperm plasma membrane due to insufficient production of GSH. Therefore, reduction in the antioxidant factors may explain sperm anomalies, such as abnormal mid-piece and decreased sperm motility in the envenomed group, as observed in this study. These factors are known to cause infertility in males, thus substantiating the ability of *E. ocellatus* venom to induce male reproductive system dysfunction in envenomed victims. Moreover, *E. ocellatus* venom-induced OS in vital organs of envenomed rats has been reported [33]. However, the mechanism of the venom toxins inducing OS is not yet clear and needs further investigation.

In addition, studies have reported that induction of sperm abnormality by toxicants may be a result of generated hydroxyl radicals (OH-) interacting with DNA in the sperm heads, which may wreak havoc on DNA integrity [34]. The observed venom induced-sperm abnormalities may suggest a genotoxic effect resulting from ROS. It has been established that elevated ROS generation and depleted antioxidant capabilities could result in sperm DNA fragmentation, either by direct or indirect effects, via the stimulation of sperm caspases and endonuclease generation [35,36]. Consequently, apoptosis may occur resulting from DNA fragmentation induced by ROS. Likewise, disruption of the mitochondrial membrane attributed to an upsurge in ROS production could trigger the release of cytochrome-C signaling molecule, which may activate apoptotic caspases and annexin-V phosphatidylserine binding activity [37]. Elevated cytochrome-C release in the seminal plasma of infertile men may result in significant damage to the mitochondria [38].

Li et al. [39] has established that the production of sperm and processes are managed by a complex-regulation of the neuroendocrine hypothalamic-pituitary gonadal axis in concert with local testicular steroids. Reproductive hormones, such as testosterone, LH, and FSH, which are male sex hormones, play a crucial role in spermatogenesis. In this study, it was observed that *E. ocellatus* venom caused a significant increase in levels of testosterone, FSH, and LH, suggesting that the venom toxins may have an impact on the biological process of steroid hormone production in the testis, thereby interfering with the hypothalamic-pituitary-gonadal axis [39]. A significant elevation of testosterone concentration in the serum of envenomed rats may arise due to an increase in genes that are liable for testosterone biosynthesis [39]. Furthermore, harmful feedback mechanisms on the hypothalamus and pituitary gland may cause a rise in testosterone concentration, leading to Leydig cell dysfunction resulting in spermatogenesis disorder [40]. On the other hand, FSH is an important hormone that plays a crucial role in testicular development combined with sperm production. However, a significant increase in concentration of serum FSH is a dependable baseline for main testicular failure, low sperm count, zero sperm in semen, and genetic inborn error [41]. In this study, significant upregulation of FSH in serum of envenomed rats may indicate damage associated with the germinal cell [42]. This result suggests that the venom could induce male sex hormonal imbalances, thereby disrupting the process of spermatogenesis and ultimately resulting in male infertility. However, the mechanism of the venom inducing hormonal dysregulation is not yet clear. 

Cytokines are small proteins secreted by the immune cells, and a moderate amount of these proteins is required to maintain the physiological functions of cells inside the testes [16]. Sertoli or spermatogenic cells produce a cautious amount of inflammatory cytokines, such as TNF-α and IL-1β, during the maturation cycles of the seminiferous epithelium, an indication they play a crucial role in controlling this basic attribute of testicular functions [43]. Detection of cytokines in typical seminiferous tubule cross-sections of the adult testis reflects their temporally regulated synthesis and function. They serve important roles in regulating steroidogenesis and immunoregulation while also contributing to pathophysiology and detrimental effects of inflammatory responses on testicular functions [43,44]. In this present study, *E. ocellatus* venom upregulated levels of IL1-β and TNF-α in testis and epididymis of the envenomed rats, suggesting that toxins in the venom can modulate pro-inflammatory cytokine release, which corroborated our earlier findings on elevated IL1-β and TNF-α in vital organs following *E. ocellatus* envenomation [33]. Studies have reported that increase in production of pro-inflammatory mediators, most especially IL1-β and TNF-α, could trigger elevated ROS formation and activate inflammatory processes, which have consequent damaging effects on normal functions of the testicular cells, particularly Sertoli and Leydig cells [45,46]. Furthermore, findings have established that up-regulation in responses of some specific inflammatory cytokines is an indication of initiated testis pathologies and revealed that significant high levels of transcripts encoding pro-inflammatory cytokines, such as IL1-β and TNF-α, are responsible for pathologies in testis cancer samples [47]. Reports from other related studies have detailed that elevated release of IL1-β and TNF-α induces inflammatory lesions, disrupts spermatogenesis [48], or causes other pathophysiological diseases [49].

A well-established report has revealed that tissue damage could occur following alteration in intracellular components, proteins, nucleic acid, and lipids due to an upsurge in ROS production in organ cells [50]. Histopathological examination revealed evidence of gross lesions in the testis tissues and inflammatory response while cauda inflammation, atrophy of tubules, and accentuation of interstitium were noticed in the epidermis of the envenomed rats. These are signs of tissue degeneration and evidence of organ toxicity due to the venom’s action on the male reproductive organs of the envenomed rats. This could probably be a prerequisite for the observed sperm anomalies produced by the defective testes. The observed pathologies on reproductive organs of envenomed rats further substantiated our previous reports that *E. ocellatus* venom is capable of inducing histological alterations on vital organs after envenoming [33]. The reproductive organ defects observed in envenomed rats in this study may have been significantly influenced by the action of the venom toxins through the induction of OS. It should be noted that OS has been implicated in cellular damage and chronic diseases, resulting in malfunctioning of reproductive functions of male organs with harmful effects on male fertility [5]. 

Finally, studies have established the mechanism underlying OS-induced male reproductive dysfunction and reported that OS alters sperm parameters with damaging effects on their structures and functions through actuated interconnected processes with LPO production of the sperm membrane, spermatozoa intracellular oxidative damage, altered sperm DNA, and apoptotic pathway activation in the germ cells [35], and these represent the underlying mechanisms of action that may be applicable to our findings in this current study. 

## 4. Conclusions

This current study has demonstrated that *E. ocellatus* venom has the intrinsic ability to induce reproductive toxicity and disrupt male reproductive functions in envenomed rats. Findings from this study indicate that toxins in *E. ocellatus* venom have the potential to incapacitate viable sperm cells, disrupt sperm production, and cause damage to the testis, such as injuries to seminiferous tubules, degeneration of Leydig cells, and necrosis of spermatogenic cells, combined with endocrine disruption. Without any doubt, these toxic effects resulting from oxidative stress inflicted on the male reproductive organs could cause various reproductive anomalies in envenomed victims. This assumes significance and is a public health concern considering the rising number of snakebite incidences in rural communities of Africa, particularly in Nigeria. Therefore, findings from this present study will draw more consciousness towards the effective treatment of snakebite envenoming to prevent systemic toxicity that could cause reproductive dysfunction resulting in male infertility, most especially in rural communities where there are challenges in accessing effective antivenom treatment. 

## 5. Materials and Methods

### 5.1. Chemicals and Kits

Rat enzyme-Linked Immunosorbent Assay (ELISA) Kits for FSH, LH, and Testosterone assays were purchased from Bio-Inteco Diagnostic, UK Ltd., Ken House, London, UK. Enzyme-linked immunosorbent assay (ELISA) kits manufactured by PeproTech, Inc., London, UK were used for TNF-a and IL-1ß assays. The reagents and chemicals were of good grade and procured from Sigma-Aldrich, St Louis, MO, USA.

### 5.2. Procurement of Snake Venom

A lyophilized sample *E. ocellatus* venom was procured from herpetarium of the Department of Pharmacology and Toxicology, Amadu Bello University, Zaria, Nigeria. The venom sample was transported at a temperature of 4 °C to the Animal Physiology Laboratory, Department of Zoology, University of Ibadan, Nigeria. The lyophilized venom was stored at 4 °C in the laboratory until use.

### 5.3. Experimental Rats 

Twenty male albino Wistar rats weighing between 180 and 220 g used for this study were obtained from the animal breeding unit of the Department of Zoology, University of Ibadan, Nigeria. The rats were kept in well-ventilated, transparent plastic cages at temperature 27 °C and the maximum number of rats in each cages was five. The animals were fed with standard rat pelletized feed and given water ad libitum. The experimental protocols were approved with assigned number UI-ACUREC: 18/0108 by the University of Ibadan-Animal Care and Research Ethics Committee (UI-ACUREC) and their guidelines were strictly followed. All animal experiments complied with the National Research Council’s publication on guide for the care and use of laboratory animals [51].

#### 5.3.1. Study Design

The experimental rats were randomly divided into two groups of ten rats (n = 10) each. Rats in group 1 served as the control and were injected with 0.2 mL of saline while rats in group 2 were envenomed with 0.2 mL of *E. ocellatus* venom. 

#### 5.3.2. Envenoming Procedures

Previous studies from our laboratory have shown the lethal dose of *E. ocellatus* venom to be 0.22 mg/bodyweight [33]. In this current study, the envenomed rats were injected intraperitoneally with 0.055 mg/kg^−1^ (LD_6.25_) of *E. ocellatus* venom to reduce fatalities to the lowest minimum. The rats were envenomed at 8:00 am on day 1 and injected with a repeated dose in the morning on day 25. The duration of the experiment and monitoring of envenomed animals was fifty days to allow the completion of spermatogenesis [52]. The control rats were injected with 0.2 mL of saline water. The experimental animals were monitored twice daily for clinical signs of toxicity, morbidity, and mortality. 

#### 5.3.3. Body Weight Determination

Prior to venom injection, the animals were weighed on the first day for their initial body weight and before they were sacrificed as terminal weight. The body weight gain was calculated using the formula:

$$Body\;weight\;gain=\frac{Terminal\;weight\;of\;mice-Initial\;weight\;of\;mice}{Initial\;weight\;of\;mice}\times 100$$

#### 5.3.4. Collection of Blood and Organ Sample

Post-exposure, blood samples were collected from the experimental rats through retro-orbital sinus punctuation using heparinized capillary tubes into plain bottles and centrifuged at 380 g for 10 min to obtain serum for hormonal assays. Rats were thereafter sacrificed through cervical dislocation following guides [53]. The cauda epididymis was surgically removed for sperm parameters analysis and the testes was removed and weighed. A portion of the epididymis and testes tissues was used for biochemical analysis and histological examination. The relative testes weight was determined using the formula:

$$Relative\;organ\;weight=\frac{Organ\;weight\;}{Termimal\;body\;weight\;\;}\times 100$$

### 5.4. Epididymal Sperm Parameters

The cauda epididymis was placed individually in a Petri dish and minced in normal saline (1 mL) to form sperm suspension, and 10 µL were placed in triplicate on microscopic slides and observed for motility under the light microscope at a magnification of ×400. Sperm motility was assessed by classifying 200 spermatozoa into two categories, motile and immotile spermatozoa. Three sperm classes were categorized as motile spermatozoa: rapid progressive, slow progressive, and nonprogressive spermatozoa [54]. To evaluate the sperm volume, the epididymis was immersed in 5 mL normal saline in a measuring cylinder and the volume displaced was taken as the volume of the epididymis. For the sperm count, a 1:10 dilution from the sperm suspension was made in a Petri dish. Spermatozoa from the left and right cauda were counted using the improved Neubauer hemocytometer. Each sample was counted three times and averaged. The sperm morphology assay was carried out according to Wyrobek et al. [55]. Sperm suspension was mixed with 1% aqueous eosin Y and smeared on microscopic slides. Abnormalities were observed in 250 spermatozoa with four replicates in each mouse at a magnification of ×1000. 

### 5.5. Hormonal Assays

The sera obtained were analyzed to determine the concentration of testosterone, follicle stimulating hormone (FSH), and luteinizing hormone (LH) using the Enzyme-Linked immunosorbent Assay (ELISA). The ELISA kits used were manufactured by Biocheck, South San Francisco, CA, USA.

### 5.6. Oxidative Stress Parameters in Testes and Epididymis

#### 5.6.1. Measurement of Catalase (CAT) Activity 

Catalase activity was determined by measuring the rate of hydrolysis of H_2_O_2_ at 240 nm [56]. Briefly, Hydrogen peroxide (8.8 mM) in sodium phosphate buffer (0.1 M, pH 7.0) was added to 0.05 mg protein of tissue samples. The decrease in absorbance was monitored for 3 min and the activity was expressed as l mol H_2_O_2_ decomposed/min/mg protein (€−43.6/mM/cm).

#### 5.6.2. Measurement of the Reduced Glutathione (GSH) Level

Levels of glutathione were measured as described [57]. GSH reacts with 5,5′-dithiobis (2-nitrobenzoic acid) (DTNB) or Ellman’s reagent to generate 2-nitro-5-thiobenzoic acid and glutathione disulphide (GSSG), a yellow-coloured compound measured spectrophotometrically at 412 nm.

#### 5.6.3. Determination of Lipid Peroxidation

Induction of oxidative damage was ascertained by measuring the extent of lipid peroxidation (LPO) in the tissue sample using estimated thiobarbituric acid reactive substances (TBARS) [58]. An aliquot of the sample (1.0 mg protein) was added to tubes containing 1.5 mL of acetic acid (pH 3.5, 20% *v*/*v*), SDS (8% *w*/*v*, 0.2 mL) and 1.5 mL thiobarbituric acid (0.8% *w*/*v*). The mixture was then heated in a boiling water bath for 45 min. The adducts formed were extracted into 1-butanol (3 mL) and the absorbance of TBARS formed was taken at 532 nm. 

### 5.7. Cytokines Responses in the Testis and Epididymis

#### 5.7.1. Preparation of Tissues Samples

Sample of frozen testis and epididymis tissues were homogenized in a 1.5 mL RIPA buffer (25 mM TrisHCl, 150 mM NaCl, 1% NP-40, 1% sodium deoxycholate, 0.1% SDS pH = 7.6) supplemented with Protease inhibitors at 4 °C. The homogenate was incubated on ice for 30 min and then centrifuged at 10,000× *g* for 30 min at 4 °C. Following centrifugation, the supernatants were transferred to a labelled Eppendorf and stored at −80 °C for cytokine measurement.

#### 5.7.2. Measurement of Tumor Necrosis Factor-Alpha (TNF-α) and Interleukin1-Beta (IL-1β) Production

Quantitative measurement of the level of cytokines was performed using Mini Enzyme Linked Immunosorbent Assay (ELISA) Development Kits (Peprotech). Well plates were set up according to the manufacturer’s instructions and read using an ELISA plate reader at 405 nm with 650 nm as the correction wavelength. Concentrations (pg/mL) of TNF-α and IL-1β in testis and epididymis were estimated respectively [59].

### 5.8. Histological Evaluation

Histology of the testis and epididymis was assessed using standard laboratory procedures. The testicular and epididymis tissues of the rats were fixed in Bouin’s solution, embedded in paraffin, sectioned into 4 µm thicknesses, and placed on microscopic slides. Slides were observed using the light microscope at a magnification of ×400 after staining with haematoxylin and eosin (H & E). 

### 5.9. Statistical Analysis

Data were analyzed using Statistical Package for Social Sciences software produced by IBM Corp. Ltd., IBM SPSS, Version 25.0, Armonk, NY, USA. Values were expressed as mean ± standard error of mean (SEM) and analyzed using a *t*-test to compare the significant (*p* < 0.05) differences between the control and test group. An independent sample test was used for comparison. 

## Figures and Tables

**Table 1:** Incidence of mortality in the envenomed group during the fifty days experimental period.

Groups.	Envenomation	Day 1	Day 19	Day 50	Mortality (%)
Control	-	-	-	-	0.00
Envenomed	-	-	1	-	10.00

Number of rats per group (n = 10).

**Table 2:** Body weight gain, testicular weight and testiculo somatic index of experimental rats.

Groups	Body Weight Gain (g)	Testicular Weight (g)	Testiculo Somatic Index (%)
Control	6.87 ± 0.83 ^b^	1.49 ± 0.06 ^b^	3.60 ± 0.56 ^b^
Envenomed	4.74 ± 0.77 ^a^	1.24 ± 0.04 ^a^	2.62 ± 0.57 ^a^

Data are represented as mean ± standard error (n = 5). Values in the same column with different superscript are considered significant (*p* < 0.05).

**Table 3:** Sperm profiles of envenomed rats.

Sperm Motility (%)			Sperm Volume (mL)	Sperm Count (10^6^/mL)
Groups	Motile	Immotile		
Control	76.89 ± 1.50 ^b^	23.11 ± 1.34 ^a^	11.00 ± 1.52 ^b^	16.64 ± 0.74 ^b^
Envenomed	18.47 ± 0.57 ^a^	81.53 ± 0.57 ^b^	6.81 ± 0.75 ^a^	7.91 ± 0.41 ^a^

Data are expressed as mean ± standard error, (n = 5). Values in the same column with different superscript are considered significant (*p* < 0.05).

**Table 4:** Sperm abnormalities.

Sperm Parameters	Control	Envenomed
Amorphous head (AM)	4.58 ± 0.47 ^a^	25.32 ± 1.09 ^b^
Banana shape (BS)	4.22 ± 0.80 ^a^	55.93 ± 1.16 ^b^
Double tails (DT)	0.00 ± 0.00 ^a^	29.10 ± 1.14 ^b^
Folded Sperm (FS)	4.21 ± 0.40 ^a^	45.80 ± 1.20 ^b^
Abnormal mid-piece (AMP)	0.00 ± 00.00 ^a^	27.47 ± 1.00 ^b^
Long and sickled hook (LSH)	2.67 ± 0.31 ^a^	28.38 ± 0.39 ^a^
Double head (DH)	0.00 ± 0.00 ^a^	21.61 ± 0.42 ^b^
Short hook (SH)	4.31 ± 0.24 ^a^	26.51 ± 1.57 ^b^
Wrong tail attachment (WTA)	2.08 ± 0.44 ^a^	39.39 ± 1.01 ^b^
Pin head (PH)	1.04 ± 0.22 ^a^	22.82 ± 0.62 ^b^
No hook (NH)	3.39 ± 0.68 ^a^	45.12 ± 1.21 ^b^
Wrong-angled hook (WAH)	5.94 ± 1.11 ^a^	40.10 ± 1.77 ^b^
Total abnormal cells	32.44 ± 0.79 ^a^	407.55 ± 2.65 ^b^
Percentage abnormalities	3.24 ± 0.77 ^a^	40.76 ± 2.50 ^b^

Data are expressed as mean ± standard error, (n = 5). Values in the same column with different superscript are considered significant (*p* < 0.05). Mean ± standard error are fractions of the 1000 sperm cells assessed.

**Table 5:** Concentrations of serum male reproductive hormones in rats envenomed with *E. ocellatus* venom.

Groups	FSH (ng/mL)	TEST (ng/mL)	LH (ng/mL)
Control	437.00 ± 28.43 ^a^	2.02 ± 0.26 ^a^	48.00 ± 1.16 ^a^
Envenomed	498.67 ± 13.86 ^b^	2.40 ± 0.09 ^b^	55.67 ± 2.02 ^b^

Data are expressed as mean ± standard error, (n = 5). Values in the same column with different superscript are considered significant (*p* < 0.05). FSH: Follicle Stimulating Hormone, TEST: Testosterone Hormone, LH: Luteinizing Hormone.
